# Exploring sources of inaccuracy and irreproducibility in the CDC bottle bioassay through direct insecticide quantification

**DOI:** 10.1186/s13071-024-06369-4

**Published:** 2024-07-19

**Authors:** Evah F. Peard, Calvin Luu, Kimberly J. Hageman, Rose Sepesy, Scott A. Bernhardt

**Affiliations:** 1 Department of Chemistry and Biochemistry, Utah State University, Logan, UT USA; 2 Department of Biology, Utah State University, Logan, UT USA

**Keywords:** Susceptibility bioassay, Dose-response curves, Lethal concentration, Chlorpyrifos, Lambda-cyhalothrin, Organophosphate, Pyrethroid, Pesticide

## Abstract

**Background:**

The Centers for Disease Control and Prevention (CDC) bottle bioassay is a commonly used susceptibility test for measuring insect response to insecticide exposure. However, inconsistencies and high variability in insect response when conducting CDC bottle bioassays have been reported in previous publications. We hypothesized that the CDC bottle bioassay results may be compromised when expected and actual insecticide concentrations in the bottles are not equivalent and that inadequate bottle cleaning and/or loss during insecticide introduction and bottle storage steps could be responsible. We explored this hypothesis by quantifying insecticides using gas chromatography tandem mass spectrometry (GC-MS/MS) in bottles that had been cleaned, prepared, and stored according to the CDC guidelines.

**Methods:**

We investigated the bottle cleaning, preparation, and storage methods outlined in the CDC bottle bioassay procedure to identify sources of irreproducibility. We also investigated the effectiveness of cleaning bottles by autoclaving because this method is commonly used in insecticide assessment laboratories. The two insecticides used in this study were chlorpyrifos and lambda-cyhalothrin (λ-cyhalothrin). Insecticides were removed from glass bioassay bottles by rinsing with ethyl-acetate and *n*-hexane and then quantified using GC-MS/MS.

**Results:**

The CDC bottle bioassay cleaning methods did not sufficiently remove both insecticides from the glass bottles. The cleaning methods removed chlorpyrifos, which has higher water solubility, more effectively than λ-cyhalothrin. Chlorpyrifos experienced significant loss during the bottle-coating process whereas λ-cyhalothrin did not. As for bottle storage, no significant decreases in insecticide concentrations were observed for 6 h following the initial drying period for either insecticide.

**Conclusions:**

The CDC bottle bioassay protocol is susceptible to producing inaccurate results since its recommended bottle cleaning method is not sufficient and semi-volatile insecticides can volatilize from the bottle during the coating process. This can lead to the CDC bottle bioassay producing erroneous LC_50_ values. High levels of random variation were also observed in our experiments, as others have previously reported. We have outlined several steps that CDC bottle bioassay users could consider that would lead to improved accuracy and reproducibility when acquiring toxicity data.

**Graphical Abstract:**

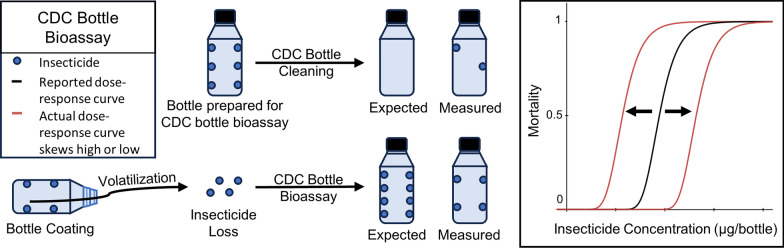

**Supplementary Information:**

The online version contains supplementary material available at 10.1186/s13071-024-06369-4.

## Background

Insect resistance to insecticides became a major topic for research after being described by Melander in 1914 [1]. The Centers for Disease Control and Prevention (CDC) bottle bioassay was developed by Brogdon and McAllister in 1998 to measure the response of adult mosquitos to specific insecticide concentrations (dose-response assessment) and test for insecticide resistance [2]. Although the CDC bottle bioassay was designed to test insecticide resistance in mosquitoes, it can also be used to test insecticide efficacy on other insect species [3, 4]. Bioassay methods are used to determine the lethal dose to 50% of test organisms (LD_50_) or the exposure concentration that kills 50% of test organisms (LC_50_) in the case of the CDC bottle bioassay. The concentration that kills 100% of insects within a 30–60-min window, known as the diagnostic dose, is also regularly measured to determine susceptibility and resistance in field populations. The CDC bottle bioassay was presented as an alternative to the World Health Organization (WHO) tube bioassay, which was developed in 1958 and involves a test kit containing tubes lined with filter papers impregnated with specific insecticide concentrations [5]. While both bioassay methods are widely used, the CDC bioassay is often preferred because of the affordability and accessibility of necessary supplies and the flexibility to test a wide range of insecticide concentrations [2, 6]. Local access and portability of materials allow for the CDC bottle bioassay to be widely used, including in lower income countries [6–12].

Researchers have previously investigated the efficacy of the CDC bottle bioassay using insect response data [7, 12–18]. Results produced from the CDC bottle bioassay and the WHO tube bioassay have also been compared with poor agreement [19, 20], good agreement [14, 21], and intermediate agreement [22–25] all being reported [13]. Moreover, researchers have concluded that both bioassays produce inconsistent dose-response curves. The CDC bioassay has been shown to produce higher random variation and more inconsistent dose response curves than the WHO tube bioassay; however, the WHO tube bioassay tends to have lower sensitivity [15, 18]. Owusu et al. suggested the need for a new standardized bioassay that produces more consistent dose-response measurements [13]. In 2022, the WHO bottle bioassay was developed as a modified version of the CDC bottle bioassay for insecticides that, due to their physicochemical properties, cannot be impregnated into filter paper [26, 27]. However, since the WHO bottle bioassay is similar to the CDC bottle bioassay, random variations and inconsistencies are also likely to occur.

We hypothesized that aspects of the CDC bottle bioassay methods for bottle cleaning, insecticide introduction, and storage can result in prepared bottles containing highly variable and incorrect insecticide concentrations. While previous investigations into the efficacy of the CDC bottle bioassay utilized insect response data, no published reports describe using measured insecticide concentrations in prepared bottles for such evaluations. To unravel the sources of inconsistencies and variability previously observed in CDC bottle bioassay data, we quantified insecticides in bioassay bottles using gas chromatography tandem mass spectrometry (GC-MS/MS) following each step in the bottle preparation process. We were particularly interested in determining if (i) the cleaning methods adequately removed insecticides from previously used bottles and (ii) insecticide volatilization and/or degradation occurred during the bottle rolling and storage steps. We used two insecticides to evaluate the CDC bottle bioassay methods: chlorpyrifos (CAS 2921-88-2), an organophosphate insecticide, and λ-cyhalothrin (CAS 91465-08-6), a pyrethroid insecticide. In this study, chlorpyrifos was selected to represent insecticides with relatively high vapor pressures and low photodegradation rates (in this case the vapor pressure is 2.69E-03 Pa [28] and the photodegradation rate (*k*) is 1.37E-03 h^-1^ (at 1000 W/m^2^) [29]), whereas λ-cyhalothrin represents those with relatively low vapor pressures and high photodegradation rates (in this case 4.47E-07 Pa [30] and 0.042 h^-1^ (at 550 W/m^2^), respectively [31]). Comparable photodegradation rates on glass surfaces for these insecticides have not been reported; therefore, the values provided are foliar photodegradation rates. In addition, both insecticides are commonly used in agriculture and tested with the CDC bottle bioassay.

## Methods

### Materials and chemicals

Optima-grade solvents (acetone, ethyl acetate, and *n*-hexane) were purchased from Thermo Fisher Scientific (Waltham, MA, USA). 1000-ml glass Wheaton bottles were purchased from Thermo Fisher Scientific and VWR International (Radnor, PA, USA). Chlorpyrifos active ingredient and the labeled standard, d10-chlorpyrifos, were purchased from Sigma-Aldrich, Inc. (St. Louis, MO, USA). λ-Cyhalothrin active ingredient was purchased from Chem Service, Inc. (West Chester, PA, USA), and the labeled standard, d5-terbuthylazine, was purchased from CDN Isotopes Inc. (Quebec, Canada). The physicochemical properties of chlorpyrifos and λ-cyhalothrin are displayed in Table 1 [28, 30, 32].

**Table 1:** Physicochemical properties of chlorpyrifos [26] and λ-cyhalothrin [28], including the equilibrium octanol–air and octanol-water partition coefficients (*K*_oa_ and *K*_ow_, respectively)

Property (at 25 °C)	Chlorpyrifos	λ-Cyhalothrin
Molecular Weight (g/mol)	350.6	449.8
Log *K*_oa_	9.81	13.03
Log *K*_ow_	4.96	7.00
Vapor pressure (Pa)	2.69E-03	4.47E-07
Decomposition temperature (°C)	160	275

All values obtained from PubChem [26, 28] except *K*_oa_ values, which were obtained from the UFZ-LSER database [30]

### Validated bottle cleaning method

All glass Wheaton bottles were cleaned with soap and tap water five times each and then rinsed with deionized water five times. Bottles were then baked for 90 min at 565 °C, which exceeds the decomposition temperatures for both chlorpyrifos (160 °C [28]) and λ-cyhalothrin (275 °C [30]). We also confirmed that insecticide concentrations in bottles cleaned with this method were below our detection limit of 1.5 ng/bottle (0.005 ng/µl) with GC-MS/MS.

### Evaluation of CDC and alternative bottle cleaning methods

To evaluate the CDC cleaning methods, we prepared bioassay bottles according to the CDC protocol [2], followed the CDC bottle cleaning steps, and then quantified remaining insecticide in the bottles. Two active-ingredient insecticide concentrations were selected that represent a typical range used in CDC bottle bioassay experiments. Nine 1000-ml glass Wheaton bottles were first cleaned following the Validated Bottle Cleaning Method. The CDC protocol suggests using 250-ml bottles; however, larger bottles are also often used [33, 34]. Following methods used in Denlinger et al., we used 1000-ml bottles and compensated for the larger bottle size by adding four times the insecticide mass that would have been used for 250-ml bottles, thus maintaining an equivalent insecticide concentration per bottle [33, 34]. A 1000-µl RAININ pipette was used to spike bottles with 4 ml of one of the following solutions prepared in acetone: low chlorpyrifos (1.020 µg/ml), high chlorpyrifos (46.37 µg/ml), low λ-cyhalothrin (1.056 µg/ml), and high λ-cyhalothrin (46.39 µg/ml).

After insecticide spiking, bottles were tilted to coat bottle and cap interior surfaces [2] and then rolled on a benchtop mechanical roller (Bellco 10 Position Cell Production Roller Apparatus) in a fume hood for 30 min [34]. During this time, the caps were slowly loosened to allow the acetone to evaporate. Once the cap was completely removed, bottles were left in the fume hood to dry completely for 24 h. The laboratory lights were off during this time to limit insecticide photodegradation. Prepared bottles were then cleaned following the CDC bioassay bottle cleaning methods by washing bottles three times with soapy water and rinsing three times with tap water [2]. Afterwards, the protocol indicates that bottles can either be air-dried or baked at 50 °C for 20 min or until dry. We compared remaining insecticide in triplicate sets of baked and air-dried bottles. The laboratory oven we used could not be set as low as 50 °C, so the actual oven temperature was 72 °C. It took 80 min for baked bottles to completely dry in the oven and 3 days for the air-dried bottles to completely dry. We also measured remaining insecticide in autoclaved bottles since autoclaving is a common method used to sterilize glassware. A triplicate set of bottles was autoclaved using a Primus steam sterilizer (Primus Sterilizer Co., Omaha, NE, USA) on a gravity cycle at 121 °C for 25 min with a 5-min dry time, which is a typical cleaning method for dry glassware.

Percent insecticide remaining in bottles was calculated using the following equation:

1 $$Percent\,Remaining = \frac{insecticide\,mass\,in\,the\,bottle\,after\,cleaning }{{insecticide\,mass\,spiked\,into\,the\,bottle}}*100$$

### Evaluation of CDC bottle preparation method

To evaluate the CDC bottle preparation methods, we prepared bioassay bottles according to the CDC protocol [2] and then quantified insecticide concentrations in the prepared bottles. Three 1000-ml glass Wheaton bottles were spiked with 4 ml of the low concentration chlorpyrifos spiking solution described above using a 1000-µl RAININ pipette. A fourth bottle was spiked with 4 ml of pure acetone to serve as a control. Bottles were then coated with insecticide and rolled on a mechanical roller for 24 h, using the previously described methods. This procedure was repeated with the high chlorpyrifos, low λ-cyhalothrin, and high λ-cyhalothrin spiking solutions, with an acetone control included with each set.

Percent loss of spiked insecticides was calculated using the following equation:

2 $$Percent \, Loss \, = \, \left( {1 - \frac{insecticide\,mass\,in\,the\,bottle\,after\,preparation }{{insecticide\,mass\,spiked\,into\,the\,bottle}}} \right)*100$$

### Evaluation of CDC bottle storage recommendation 

The CDC bottle bioassay protocol includes a recommended storage procedure for when bioassays are not performed immediately after bottle preparation. The recommended method is to store prepared bottles in a dark place with their caps off for 12 h to 5 days [2]. We evaluated this method by preparing bioassay bottles, storing them for 6 h, and then quantifying the remaining insecticide in the bottles. Nine bottles were cleaned with the Validated Cleaning Method and then spiked with 4 ml of the high chlorpyrifos spiking solution and coated according to the previously described method. The insecticide in three of the bottles was quantified directly after the 24-h rolling process, while the other six bottles were stored in a dark cabinet with the caps off until insecticide quantification. Three of the remaining bottles were stored for 3 h, and the other three were stored for 6 h. This process was repeated using the high λ-cyhalothrin spiking solution.

### Insecticide quantification

To quantify insecticide concentrations in prepared CDC bioassay bottles, we rinsed prepared bottles with solvents and then quantified the insecticide in rinsates using GC-MS/MS. Each prepared glass bottle was rinsed twice with 20 ml each of ethyl acetate and *n*-hexane solvents, which were combined. An automated solvent evaporator system (Biotage Turbovap® II) was used to concentrate the rinsates to 300 µl. The nitrogen flow rate was set to 6.5 l/min, and the water bath temperature was 35 °C. Concentrated rinsates were transferred into 2-ml GC vials containing 400-µl glass inserts. We also confirmed the concentration of the spiking solutions by adding 4 ml of each insecticide spiking solution to individual Turbovap® tubes with ~ 5 ml of both ethyl acetate and *n*-hexane and reduced the volume to 300 µl according to the previously described methods. A combination of 20 ml of ethyl acetate and *n*-hexane was concentrated to serve as a laboratory blank. Finally, two 50 ml acetone blanks were also prepared, one from the solvent bottle used in the GC-MS/MS laboratory and one from the bottle preparation laboratory. These were blown down to 300 µl and transferred to GC vials following the previously described methods.

Samples that were expected to have an insecticide concentration exceeding the calibration range (>20 ng/µl) were diluted in a 1:50 ratio by taking 6 µl of the sample using a Hamilton syringe and diluting to 300 µl with ethyl acetate. Other samples and the blanks were not diluted. Samples and blanks were spiked with an isotopically labeled internal standard to monitor instrument performance. All samples that contained chlorpyrifos were spiked with 20 µl of 8 ng/µl d10-chlorpyrifos internal standard using a Drummond syringe. All samples that contained λ-cyhalothrin were spiked with 20 µl of 8 ng/µl d5-terbuthylazine internal standard. All blanks were spiked with 20 µl of both internal standard solutions. Samples were then stored at 4 °C until analysis. All samples were analyzed within 2-3 weeks.

Insecticides were quantified using a Thermo Fisher Scientific (Waltham, MA, USA) Trace 1310 GC and a TSQ 8000™ Evo triple-quadrupole MS. Target analytes were separated with a 30 m × 0.25 mm × 0.25 µm ZB-5MSplus fused silica capillary column (Phenomenex, Torrance, CA, USA) with a 10-m deactivated guard column (Thermo Fisher Scientific). Helium was used as the carrier gas at a column flow rate of 1.2 ml/min. The inlet temperature was 300 °C, and injections were conducted in splitless mode. The GC oven temperature was initially 70 °C (held for 0.66 min), ramped by 50 °C/min to 150 °C, ramped by 6 °C/min to 200 °C, ramped by 16 °C/min to 300 °C, and held for 6 min. The MS was operated in selective reaction monitoring (SRM) mode. Target analyte retention times and SRM transitions are provided in Additional file 1: Table S1. An internal 10-point calibration curve with a calibration range of 0.005–20 ng/µl was prepared for both chlorpyrifos and λ-cyhalothrin using the ratio of the target analyte peak area to the corresponding internal standard peak area. Additionally, a calibration verification standard was run after every six to nine samples to monitor instrument variability and calibration curve robustness.

### Statistical analysis

All statistical analyses were performed using the statistical program R v4.3.2 [35]. Normality and homogeneity of variance were tested using a Shapiro-Wilk test and Bartlett’s test, respectively. The low concentration data for both chlorpyrifos and λ-cyhalothrin did not display a normal distribution (Shapiro-Wilk, *p* < 0.05). Each data set in the bottle cleaning evaluation had unequal variance (Bartlett’s test, *p* < 0.05). To overcome these issues with normality and homogeneity of variance, we performed a log transformation on the data before performing analysis of variance (ANOVA) tests to compare the three bottle cleaning methods (baking, air-drying, and autoclaving) at low and high concentrations for both chlorpyrifos and λ-cyhalothrin. If the ANOVA test indicated statistical significance (*p* < 0.05), Tukey’s honestly significant difference (Tukey’s HSD) tests were performed. For the evaluation of the bottle coating process, the Shapiro-Wilks test (*p* > 0.05) indicated that the data were normally distributed and the Bartlett’s test (*p* > 0.05) indicated that the variance was equal. Therefore, two-sample t-tests (assuming equal variance) were performed to compare the percent insecticide loss during bottle coating using low and high concentrations for both chlorpyrifos and λ-cyhalothrin. The percent loss during the bottle storage evaluation was analyzed using ANOVA tests, and Tukey’s HSD was performed to compare the different storage times (0, 3, and 6 h) for both chlorpyrifos and λ-cyhalothrin. The bottle storage evaluation results were normally distributed (Shapiro-Wilk *p* > 0.05) for both chlorpyrifos and λ-cyhalothrin. However, the λ-cyhalothrin storage data had unequal variance (Bartlett’s test, *p* < 0.05) that was not improved with a log transformation. Therefore, the ANOVA was performed on the untransformed bottle storage data.

## Results and discussion

### Evaluation of CDC and alternative bottle cleaning methods

**Fig. 1:**
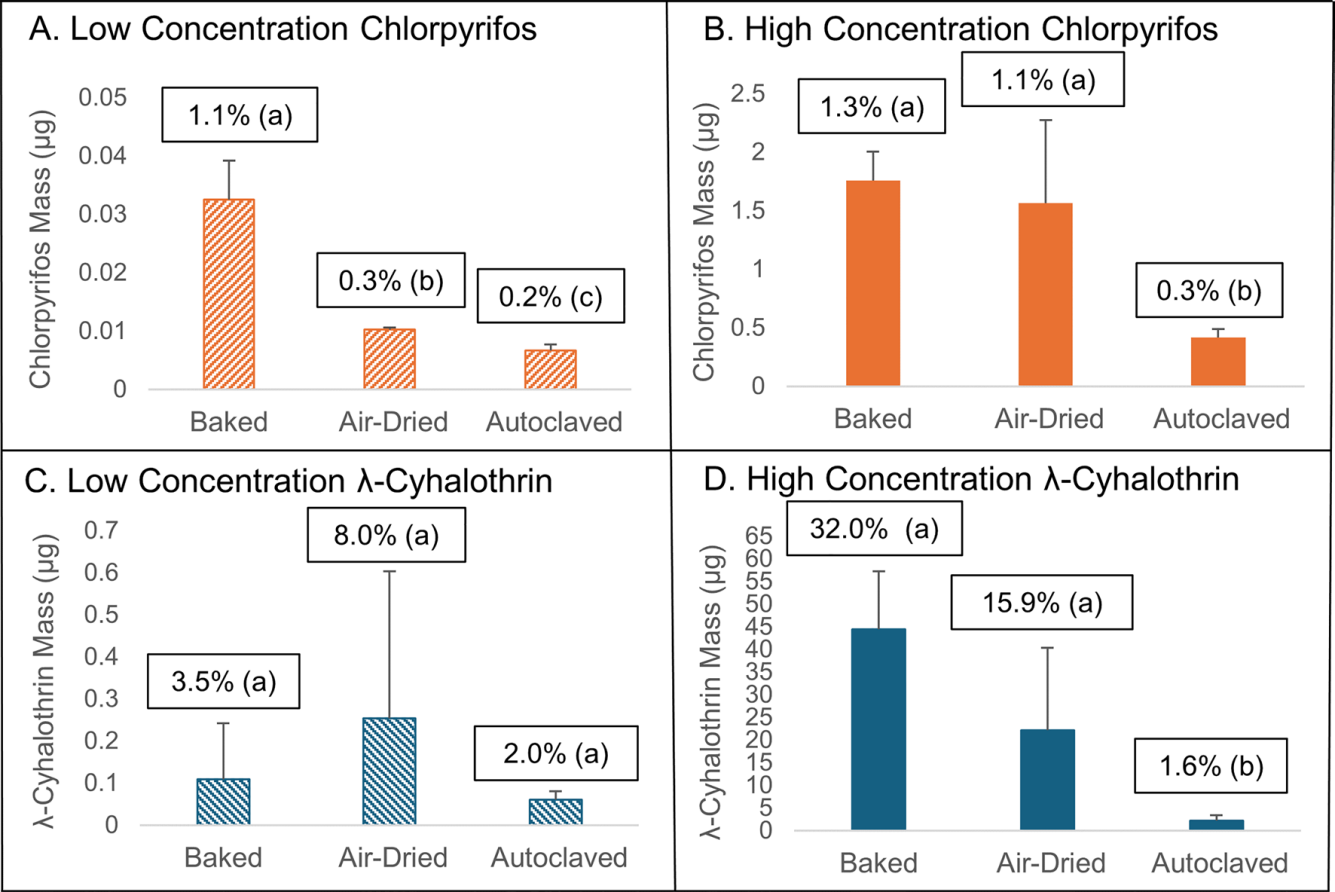
Mean insecticide mass remaining (± SD, *n* = 3) for chlorpyrifos (**A** low;** B** high) and λ-cyhalothrin (**C** low;** D**: high) following the CDC recommended cleaning methods based on baking and air-drying or using an autoclave. Mean insecticide percentages remaining in cleaned bottles are provided above bars. Letters in parentheses above bars denote statistical significance (Tukey’s HSD, *p* < 0.05); when letters are the same in a panel, means are not significantly different. Note the difference in axis scaling

The mean insecticide masses remaining in bottles following the two CDC cleaning methods (baking and air-drying), as well as the autoclave method, are shown by the bars in Fig. 1 (and in Additional file 2: Table S2). The percent insecticide remaining, based on spiked amounts in bottles (Eq. 1), is displayed above each bar. For chlorpyrifos, the percent remaining in bottles after cleaning was < 1.5% in all cases, meaning that all cleaning methods removed at least 98.5% of the spiked chlorpyrifos (Fig. 1A, B). We expect that any chlorpyrifos lost after the washing and rinsing steps was due to volatilization because it decomposes at 160 °C [28], which was not reached in any of the tested cleaning methods, it photodegrades slowly (*k* = 1.37E-03 h^-1^ at 1000 W/m^2^, keeping in mind that this is the rate on leaf surfaces, not on glass [29]), and it has a high vapor pressure (2.69E-03 Pa [28]).

For the low concentration chlorpyrifos bottles, the baking method resulted in a mean residual mass of 0.03 ± 0.01 µg (mean ± SD), which was the highest mean mass remaining out of the three treatments and was thus the least effective cleaning method (Fig. 1A). The relatively high variability in percent remaining for these bottles was due to the bottle in the middle of the oven having more insecticide remaining than those placed near the edges of the oven (data not shown), likely because of spatial temperature variations within the oven. The air-dried bottles needed significantly more time to dry than the baked ones (3 days verses 80 min) and were exposed to light during the dry time, explaining the significantly lower amount of remaining chlorpyrifos after cleaning. The residual chlorpyrifos mass in the autoclaved bottles was also significantly lower than in the baked and air-dried bottles, likely because of the higher temperature in the autoclave (121 °C). While volatilization from glass surfaces would have occurred at the autoclave temperature (121 ºC), this temperature is below the chlorpyrifos decomposition temperature (161 ºC [28]).

The high concentration chlorpyrifos bottles contained higher mean residual insecticide masses in all cases compared to the low concentration chlorpyrifos bottles, with the highest mean residual masses being 1.76 ± 0.25 µg and 1.57 ± 0.71 µg in the bottles that were baked and air-dried, respectively (Fig. 1B). In contrast to that observed with the low concentration bottles, the residual masses in the baked and air-dried bottles were similar but were both significantly higher than in the autoclaved bottles. The results for the air-dried samples had high variability, likely due to differences in light exposure and air flow during the drying process.

The residual masses remaining in bottles after cleaning were used to determine whether bottle contamination was substantial enough to affect measured LC_50_ results. We assessed this by calculating the true LC_50_ from the measured LC_50_ when bottle contamination was present using Eq. 3.

3 $$True\,LC_{{50}} = Measured\,LC_{{50}} + Mean\,Residual\,Mass$$

where the true LC_50_ is based on the actual insecticide concentration that is lethal to 50% of insects. For example, consider a situation in which experiments were conducted with baked bottles that were previously spiked with the high chlorpyrifos solution and thus contained 1.8 µg/bottle residual chlorpyrifos after the cleaning process. If an LC_50_ of 10 µg/bottle was measured during a dose-response experiment, the true LC_50_ would have been 11.8 µg/bottle, resulting in a measured value that is 15% lower than the true value. The degree to which bottle contamination skews measured values worsens as the LC_50_ approaches the residual mass. For example, with the same residual mass used above, if the measured LC_50_ was 5 µg/bottle, it would be 26% lower than the true LC_50_ whereas if the measured LC50 was 100 µg/bottle, it would be too low by 2%.

The percentage λ-cyhalothrin remaining in cleaned bottles was much higher than for chlorpyrifos in all cases (Fig. 1C, D). In the most extreme case, the mean percent remaining was 32.0 ± 9.2% for the high concentration baked bottles. λ-Cyhalothrin decomposes at 275 °C [30], which was not reached in any of the tested cleaning methods. While λ-cyhalothrin does photodegrade quickly (*k* = 0.04 h^-1^ at 500 W/m^2^ on leaves [31]) compared to chlorpyrifos (*k* = 1.37E-03 h^-1^ at 1000 W/m^2^ on leaves), it would not have been exposed to light in either the oven or the autoclave. Since λ-cyhalothrin is also not volatile (4.47E-07 Pa [30]), we expect that most of the λ-cyhalothrin that was removed from bottles occurred during the washing and rinsing steps. However, the octanol-water equilibrium partition coefficient (*K*_ow_), which can serve as a proxy for the glass-water equilibrium partition coefficient, for λ-cyhalothrin is more than 100 times higher than that of chlorpyrifos (log *K*_ow_ = 7.00 for λ-cyhalothrin [30]; 4.96 for chlorpyrifos [28], Table 1), explaining why washing and rinsing with water could leave significant amounts of λ-cyhalothrin behind.

For the low-concentration λ-cyhalothrin bottles, there were no statistical differences between results for the three cleaning methods, with mean residual masses of 0.11 ± 0.13 µg, 0.25 ± 0.35 µg, and 0.06 ± 0.02 µg for the baked, air-dried, and autoclaved treatments, respectively (Fig. 1C). However, baking and air-drying resulted in high variability likely for the same reasons as mentioned above for chlorpyrifos. For the high concentration λ-cyhalothrin tests, the residual mass in the baked and air-dried bottles was significantly higher than for the autoclaved bottles, but the differences between baking and air-drying were not statistically different (Fig. 1D). Overall, autoclaving proved to be the most effective and consistent cleaning method out of the three tested. With the mean residual mass of λ-cyhalothrin reaching up to 44.5 ± 12.9 µg/bottle in the high concentration baked bottles and 22.2 ± 18.3 µg in the air-dried bottles, the likelihood that reported LC_50_ values could be skewed is high. If the residual mass was 44.5 µg/bottle and the measured LC_50_ was 5, 10, or 100 µg/bottle, then these values would be lower than the true LC_50_s by 90%, 82%, and 31%, respectively.

In our experiments, we “contaminated” the bottles only once before testing. However, it is easy to imagine this problem compounding over time in a laboratory that uses bottles multiple times. In addition, if bottles are used in tests with different insecticides, the residual of insecticide A that was used in a previous experiment may alter insect response to insecticide B used in the next experiment. In the CDC bottle bioassay method, only one control bottle is used to correct for contamination in “clean” bottles. From what we observed, it is likely that one bottle does not represent the entire set of “clean” bottles due to differences in contamination among bottles. Our results highlight the importance of using more effective bottle cleaning methods than those recommended in the CDC bottle bioassay. One option is to bake bottles at a temperature above the decomposition temperature, as we did in our Validated Cleaning Method; however, high temperature ovens are not available in most laboratories. In fact, the oven we used is a glass blower’s oven. Another option is to rinse bottles with organic solvents (e.g. acetone, methanol, *n*-hexane), as is recommended in the new WHO bottle bioassay method. Solvent rinsing is much more effective at removing small organic compounds from glass than washing with water because of their much higher solubilities in organic solvents compared to water. The WHO bottle bioassay method also recommends using Decon® cleaning solution; since it contains potassium tall oil soap and nonionic detergent, it likely also improves removal of water-insoluble insecticides from glass bottles [27].

### Evaluation of CDC bottle preparation method

**Fig. 2:**
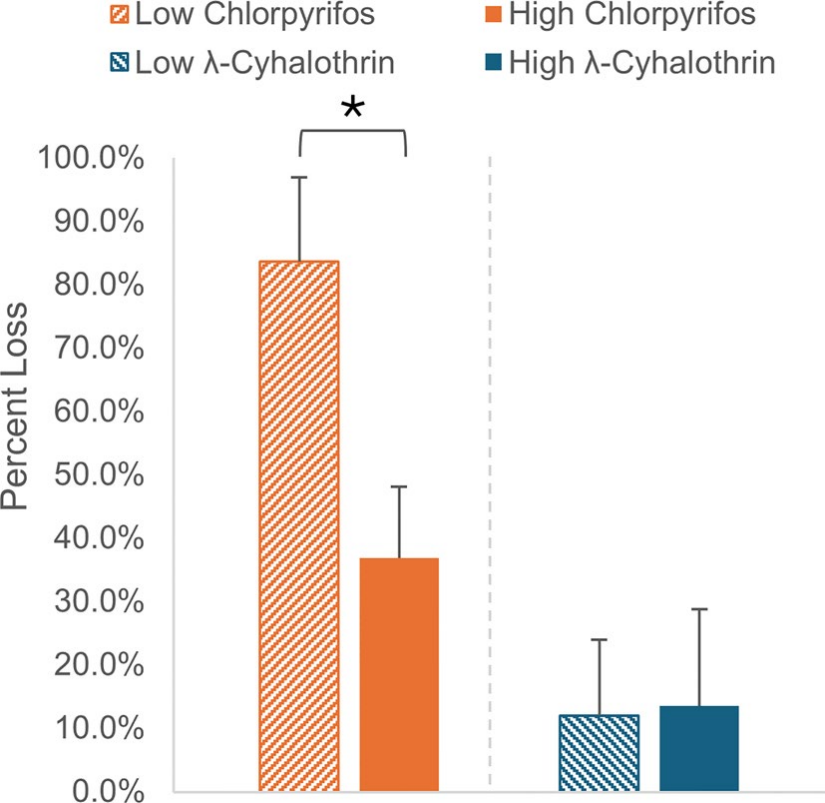
Mean insecticide percent loss (± SD, *n* = 3) after the CDC bottle coating process using low concentration and high concentration insecticide solutions of either chlorpyrifos or λ-cyhalothrin. *Difference is statistically significant (two-sample t-test, *p* < 0.05)

The mean percent insecticide loss (Eq. 2) was calculated for each set of bioassay bottles after the bottle coating process (Fig. 2, Additional file 3: Table S3). The bioassay bottles containing chlorpyrifos showed significant loss, indicating volatilization from glass during the coating process, as could be predicted from its high vapor pressure [28] and relatively low octanol-air equilibrium partition coefficient (*K*_oa_) [32] (Table 1), again using octanol as a proxy for glass. The low concentration chlorpyrifos bottles had a mean percent loss of 83.7 ± 13.3% while the high concentration chlorpyrifos bottles had a mean percent loss of 36.9 ± 11.3%. These loss percentages were significantly different, indicating that the chlorpyrifos dissipation rate from glass is concentration dependent. Further investigation is needed to fully understand this observation; however, it would occur if chlorpyrifos:chlorpyrifos intermolecular interactions are stronger than chlorpyrifos:glass interactions and there are more chlorpyrifos:chlorpyrifos interactions in the high-concentration bottles, leading to less volatilization.

The bioassay bottles containing λ-cyhalothrin had a much lower percent loss during the bottle coating process than those containing chlorpyrifos. This is understandable considering λ-cyhalothrin’s lower vapor pressure [30] and estimated low *K*_oa_ [32] (Table 1). The mean percent loss from the low and high concentration bottles was 12.1 ± 11.9% and 13.5 ± 15.3%, respectively. This indicates that, unlike chlorpyrifos, the λ-cyhalothrin dissipation from glass is not concentration dependent.

These experiments demonstrate that the actual mass of insecticide in bottles prepared for CDC bottle bioassays can be much different than the expected masses, especially for more volatile insecticides, such as chlorpyrifos. Future research could be directed at measuring percent loss for a wider range of insecticides and even developing a tool for predicting insecticide loss during bottle preparation from an insecticide’s *K*_oa_, vapor pressure, and/or other physicochemical property. The best predictions would be made from glass-air equilibrium partition coefficients; however, such values are not commonly measured and have not been measured for chlorpyrifos or λ-cyhalothrin.

Analogous to that observed with the bottle cleaning methods, significant percent loss of an insecticide during the bottle preparation procedure would result in erroneous LC_50_ reporting. For example, consider a situation in which a significant amount of chlorpyrifos was lost during the bottle coating process, leading the experimentalist to base their LC_50_ calculations on expected exposure concentrations that are much lower than actual concentrations. We used Eq. 4 to quantify the extent to which measured values would be skewed for various loss percentages.

4 $$True\,LC_{50} = \frac{{\left( {100 - Mean\,Percent\,Loss} \right) * Measured\,LC_{50} }}{100}$$

If the measured LC_50_ was 10 µg/bottle and 84% of chlorpyrifos had been lost during bottle coating, as we observed for the high concentration chlorpyrifos bottles, then the true LC_50_ would have been 1.6 µg/bottle, which is six times lower than the true amount. In contrast to that observed when considering the residual masses in bottles after cleaning, the magnitude of error is not dependent on the measured LC_50_ value. For example, if the measured LC_50_ was 100 µg/bottle, the true LC_50_ would have been 16 µg/bottle, which is still six times lower than the true amount. Since the percent loss amounts for λ-cyhalothrin during the bottle coating process were much lower than for chlorpyrifos, errors associated with measured LC_50_ values would be much lower.

### Evaluation of CDC bottle storage recommendation

**Fig. 3:**
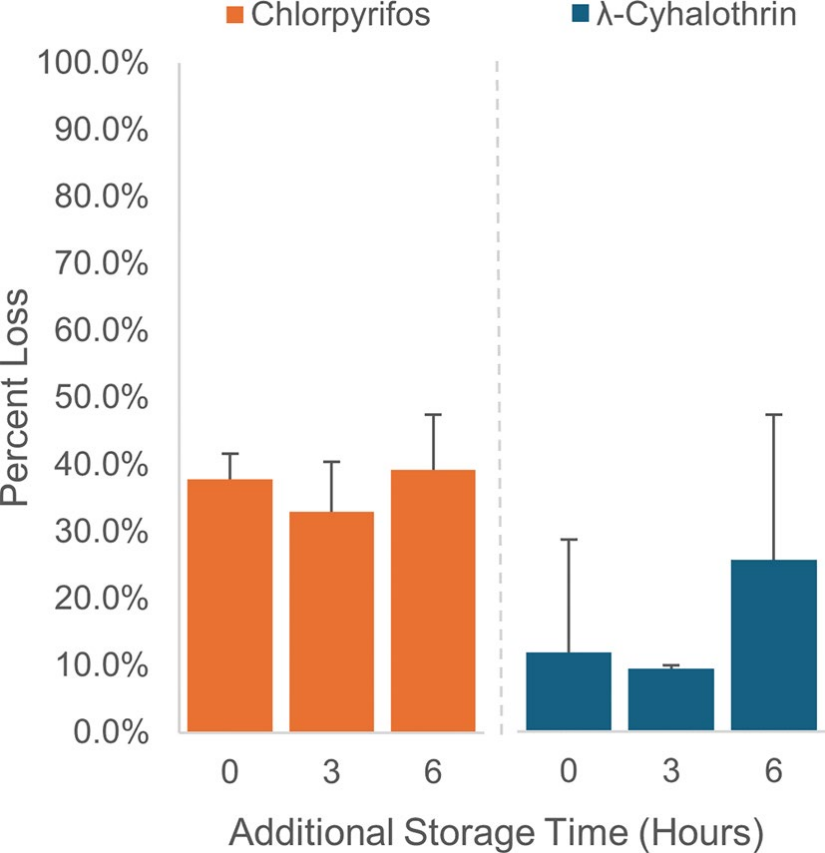
Mean percent insecticide loss (± SD, *n* = 3) for bioassay bottles prepared using high concentration spiking solution of either chlorpyrifos or λ-cyhalothrin and stored for 0, 3, and 6 h after the bottle coating process

Following the bottle coating process and then storage of bottles for 3 or 6 h, we found no significant additional loss of insecticide in the bottles for either chlorpyrifos or λ-cyhalothrin (Fig. 3, Additional file 4: Table S4). Nonetheless, future work could be directed at evaluating how insecticide dissipates in prepared bottles for periods > 6 h, especially since the CDC method states that prepared bottles can be stored from 12 h to 5 days [2]. Dissipation after the initial coating process could also vary for different insecticides and concentrations.

### Implications and recommendations

**Fig. 4:**
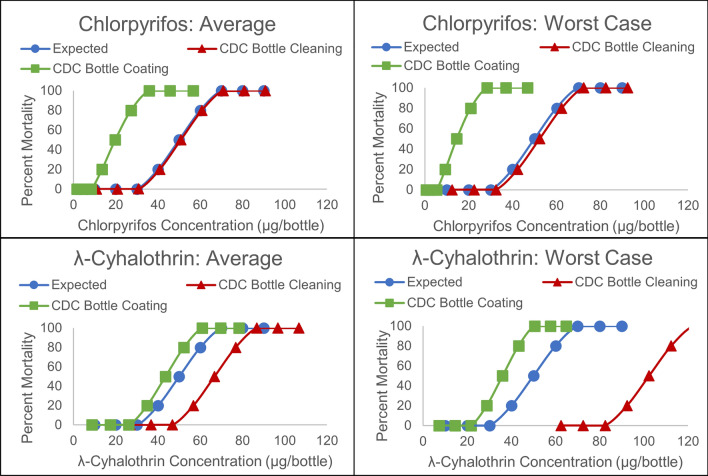
Theoretical dose-response curves for chlorpyrifos and λ-cyhalothrin based on results from the CDC bottle cleaning and coating experiments, showing calculated shifts for the average and worst-case scenarios

The results from the CDC bottle bioassay cleaning and insecticide coating experiments indicate that shifts in dose-response curves and high variability in insect response are likely to occur. Since we did not conduct bioassays in this study, we created theoretical dose-response curves to simulate how the calculated LC_50_ values would shift (Fig. 4). We created simple S-curves with theoretical insecticide concentrations and percent mortalities and then used our results from the cleaning and coating evaluations to demonstrate how expected dose-response curves would shift for chlorpyrifos and λ-cyhalothrin. The dose-response curve for chlorpyrifos was affected mainly by insecticide dissipation during the bottle coating process, which shifts the curve to the left. This could lead to an insecticide being labeled as less toxic than it truly is. Consider a scenario in which the CDC bottle bioassay was used for a beneficial insect (e.g. honeybees) and the measured LC_50_ was lower than the true LC_50_. This could be a major concern if the result of a pesticide risk assessment was a recommendation to release honeybees onto a field when the pesticide concentrations are actually lethal. In a hypothetical situation, our results indicate that if the measured LC_50_ value was 50 µg/bottle for chlorpyrifos, the true concentration in the bottle would have been 16.8 µg/bottle (considering effects from both the bottle contamination and insecticide loss during coating). In this situation, the recommendation would have been to release bees onto the field when the insecticide concentration was three times higher than the true LC_50_, which could be detrimental to honeybees. Although the CDC bottle bioassay is not commonly used with beneficial insects, this application has been used to evaluate their susceptibility to the insecticide concentrations needed to manage pests [4].

For λ-cyhalothrin, dose-response curves are more affected by inadequate bottle cleaning, which shifts the curve to the right when concentrations in bottles are higher than expected. This could result in an insecticide being labeled as more toxic than it is, leading to less insecticide being applied to control pests than is necessary. Consider a hypothetical situation in which the measured LC_50_ was 50 µg/bottle when the true concentration was 88.4 µg/bottle (as calculated from our bottle contamination and insecticide loss during coating experiments). In this situation, the recommendation would have been to apply a concentration of insecticide onto a field that is ineffective at eliminating the pests.

Our recommendations for modifying the CDC bottle bioassay include:

i. Use cleaning methods that are confirmed to remove insecticides from the glass Wheaton bottles before use. The methods outlined in the CDC methods are not sufficient. Many methods of pesticide analysis from the Environmental Protection Agency (EPA) recommend solvent rinsing glassware with the last solvent used before cleaning the glassware with soap and water [36–38]. Therefore, our cleaning recommendation is to first rinse each bottle with acetone then wash the bottles with warm soapy water and rinse with water three times. Finally, autoclave or bake the bottles at temperatures above the decomposition temperature for the insecticides in use. The EPA recommends rinsing glassware with acetone and *n*-hexane if the heating step is skipped [36–38]. Therefore, if an autoclave or oven is not available, we recommend doing additional organic solvent rinses after cleaning with soap and water, especially for less water-soluble insecticides.
ii. Avoid using the CDC bioassay with more volatile insecticides since we observed significant insecticide loss for chlorpyrifos (vapor pressure = 2.69E-03 Pa [28]), which was the more volatile of the tested insecticides in this study. On the other hand, λ-cyhalothrin (vapor pressure = 4.47E-07 Pa [30]), the less volatile insecticide in our study, showed very little loss in the bottle preparation process.
iii. Work with an analytical chemistry laboratory, if possible, to measure the efficacy of the cleaning methods, bottle coating process, and storage of the bioassay bottles. This approach can be used to identify sources of error and irreproducibility unique to the laboratory performing the bioassays and the insecticides being tested. It can also be used to determine which step of the CDC bottle bioassay introduces the largest errors.
iv. Approaches used in preparing pesticide formulations could be utilized to decrease active ingredient volatilization. The addition of adjuvants, such as surfactants, when preparing a bioassay using the CDC bioassay method could decrease the volatilization of the insecticide active ingredient. For example, high surfactant to active ingredient ratios (100:1) reduced the volatilization of applied pyrimethanil on glass surfaces by 50%, on average [39]. More studies should be done to fully understand the effect of adjuvants on active ingredient volatilization from glass.
v. Alternative approaches to determining lethal insecticide concentrations could provide more applicable results than the CDC bottle bioassay by better replicating real-world scenarios. For example, the EPA’s ‘Honeybee Toxicity Residues on Foliage’ test was designed to identify residual insecticide concentrations in foliage that are lethal to honeybees [40]. This test is easily adaptable to other insects as shown in a study designed to determine residual insecticide concentration contact toxicity on foliage for a beneficial insect parasitoid, *Diglyphus isea* [41]. Calculated lethal insecticide concentrations using the EPA guidelines account for typical environmental conditions, volatilization, degradation, and insect contact with foliage.

## Conclusions

The work presented here shows that calculated LC_50_ values determined using the CDC bottle bioassay are prone to inaccuracy in situations when re-used bottles are not adequately cleaned and/or insecticides volatilize from bottles during bottle preparation. These findings are critical, especially since the CDC bottle bioassay is commonly used to determine LC_50_ values and to test for insecticide resistance. Our work sheds new light on the factors that could possibly affect results produced from the CDC bottle bioassay. We conclude that the CDC bottle cleaning methods are not sufficient in most cases, leading to contamination that would be expected to affect calculated LC_50_ values. Insecticide loss during the bottle coating process is also likely for more volatile insecticides. In addition, we observed high levels of random variation, as others have reported when using the CDC bottle bioassay. This work provides recommendations to improve the efficacy of the CDC bottle bioassay to produce more reliable LC_50_ data. Future work should be conducted to test the efficacy of the CDC bottle bioassay with other insecticides and directly compare different methods, including the new WHO bottle bioassay, to determine lethal insecticide concentration through direct insecticide quantification. Additionally, adsorption of insecticides to glass needs to be better understood to determine how strongly different insecticides bind to glass surfaces and how easily they are desorbed. Finally, better approaches could be developed to increase insecticide adsorption to glass when performing a bottle bioassay and to increase desorption when cleaning the glass bottles.

### Supplementary Information

Additional file 1: Table S1. Target analyte retention times and selected reaction monitoring (SRM) transitions.

Additional file 2: Table S2. Insecticide mass remaining for chlorpyrifos (low and high) and λ-cyhalothrin (low and high) following the CDC recommended cleaning methods based on baking and air-drying or using an autoclave (corresponds to Fig. 1).

Additional file 3: Table S3. Insecticide percent loss after the CDC bottle coating process using low concentration and high concentration insecticide solutions of either chlorpyrifos or λ-cyhalothrin (corresponds to Fig. 2).

Additional file 4: Table S4. Percent insecticide loss for bioassay bottles prepared using high concentration spiking solution of either chlorpyrifos or λ-cyhalothrin and stored for 0, 3, and 6 h after the bottle coating process (corresponds to Fig. 3).

## Data Availability

All data generated or analyzed during this study are included in this published article and its additional files.

## Abbreviations

CDC: Centers for disease control and prevention
LD_50_: Lethal dose that kills 50% of exposed insects
LC_50_: Lethal concentration that kills 50% of exposed insects
WHO: World Health Organization
GC-MS/MS: Gas chromatography-tandem mass spectrometry
ANOVA: Analysis of variance
HSD: Honestly significant difference
EPA: Environmental Protection Agency
GC: Gas chromatograph
MS: Mass spectrometer
SRM: Selective reaction monitoring
